# Inhaled Organophosphorus Poisoning Presenting as Superior Laryngeal Nerve Palsy: A Rare Case

**DOI:** 10.7759/cureus.31589

**Published:** 2022-11-16

**Authors:** Sujal Patel, Mansi Patel, Vaidehi Hande, Shraddha Jain, Sunil Kumar, Sourya Acharya

**Affiliations:** 1 Department of Medicine, Jawaharlal Nehru Medical College, Datta Meghe Institute of Medical Sciences, Wardha, IND; 2 Department of Otolaryngology - Head and Neck Surgery, Jawaharlal Nehru Medical College, Datta Meghe Institute of Medical Sciences, Wardha, IND

**Keywords:** vocal cord palsy, intoxication, atropine, organophosphorus, inhalation

## Abstract

Poisoning by organophosphorus (OP) is a major clinical issue affecting many nations worldwide, especially developing nations. In this case report, we have highlighted organophosphate poisoning syndrome that resulted in paralysis of the vocal cords. A 28-year-old male patient with a history of accidental inhalation of the OP compound reported to our hospital with symptoms of vomiting and hoarseness of voice. He had nasal regurgitation and hoarseness having both 9th and 10th cranial nerve palsies on admission, which improved after administration of atropine.

## Introduction

Due to the widespread availability of organophosphorus (OP) compounds and over-the-counter sales, agricultural areas account for most poisoning occurrences. Decreases in acetylcholine esterase activity are typically used to validate laboratory evidence of OP poisoning.

Three unique toxicity signs of OP chemical poisoning vary depending on when they first manifest. There are three types of injuries: immediate, moderate, and delayed. A cholinergic crisis and perhaps death could result from consuming a lot of pesticides. Lesser dosages may expose people to the intermediate type or delayed type toxic syndrome [1].

Some OP esters have an uncommon hazard known as organophosphate-induced delayed polyneuropathy (OPIDP). One to four weeks after a single or brief exposure, the distant degeneration of a few axons in the peripheral and central nervous systems serves as evidence. Sometimes the delayed-type condition has a side effect of muscle weakness that lasts for a few months [2-5]. This case report highlights accidental inhalational OP compound exposure, which resulted in vocal cord palsy due to superior laryngeal nerve palsy. Probably this is the first case report of OP exposure.

## Case presentation

A 28-year-old man who claimed to have inhaled organophosphorus (Monocil 50%) while working on his farm in the Yavatmahal district of Nagpur arrived at the hospital. The patient and his family were unaware of the amount. On arrival, the general examination revealed the patient was conscious and oriented, had a pulse of 110 beats per minute, blood pressure was 100/70 mm of Hg, and saturation was 99% on room air. Cardiovascular and respiratory system examination revealed no abnormality in the central nervous system and he had a Glasgow Coma Scale score of 15/15. Laboratory parameters of the patient have been highlighted in Table 1.

**Table 1:** Laboratory investigations

Investigation	Patient’s report	Reference values
Hemoglobin	11 g/dl	13-17 g/dl
White blood cells	10.0 × 10^3^/μl	(4-10) × 10^3^/μl
Platelet count	2.1 × 10^4^/μl	(1.5-4.10) × 10^4^/μl
Serum cholinesterase	0.2 mg/dl	5.9-12.2 mg/dl
Serum creatinine	0.9 mg/dl	0.6-1.25 mg/dl
Serum urea	40 mg/dl	19-43 mg/dl
Serum sodium	148 meq/L	135-155 meq/L
Serum potassium	3.8 meq/L	3.5-5.1 meq/L
Random blood glucose	88 mg/dl	70-150 mg/dl
Total protein	7.4 gm/dl	6.3-8.2 gm/dl
Albumin	3.7 gm/dl	3.5-5.0 gm/dl
Aspartate aminotransferase	25 units/l	<50 units/l
Alanine aminotransferase	29 units/l	17-59 units/l
Alkaline phosphatase	101 IU/l	38-126 IU/l
Total bilirubin	1.2 mg/dl	0.2-1.3 mg/dl
Globulin	3.7 gm/dl	2.3-3.5 gm/dl

Following the atropinization dose, the patient was started on an 8 ml/hour injection of injectable atropine and received pralidoxime infusion at the rate of 20 ml/hour. The patient was given supportive treatments with antibiotics, proton pump inhibitors, nutritional supplements, and hydration. The infusion of atropine was gradually tapered based on clinical response. After a period of four days, atropine was started with bolus doses.

After eight days, the patient started to get hoarseness of voice, had an absence of gag reflex, and had trouble deglutinating. Muscle tone was normal. Power was 4/5 in all four limbs. Reflexes in the deep tendons revealed hyporeflexia. A bilateral plantar flexor reaction was visible. For the purpose of evaluating voice hoarseness, we sought the advice of an ENT surgeon who performed indirect laryngoscopy or video laryngoscopy. The patient's left vocal cord was discovered to be immovable (Video 1).

**Video 1:** Video-directed laryngoscopy showing the phonatory gap

Ryle's tube was placed for feeding, bolus doses of atropine were tapered off gradually, routine clinical examinations of muscle tone and power were examined regularly, and the patient began speech therapy. The quality of voice improved after seven days of speech therapy and physiotherapy was also done after which the patient regained full power of 5/5 in all four limbs and the reflex became normal. Hence, the patient was discharged with regular follow-up after a hospital stay of 15 days.

## Discussion

Organophosphate poisoning is a common reason for admission to hospitals and intensive care units in developing countries [6]. This is an illustration of an organophosphorus poisoning case with typical cholinergic symptoms on the first day and progressive involvement of the 9th and 10th cranial nerves beginning on day two. The superior laryngeal nerve supplies all laryngeal muscles except the cricothyroid muscle. This indicates that intermediate syndrome may include vocal cord palsy and that patients should receive atropine in rather high dosages. The term "intermediate syndrome" was first used in 1987 by Senanayake of Sri Lanka [4], but Wadia first used the term "type II paralysis" to describe the illness in 1974. It was given the moniker "intermediate" because its symptoms appear after the acute cholinergic phase but before the predicted onset of delayed neuropathy. The main symptoms of this condition, which typically manifest between 12 and 96 hours after consuming the toxin, include paralysis of the respiratory muscles, cranial nerve palsies, neck flexor weakness, and proximal muscular weakness.

The patient develops a combination of muscarinic, nicotinic, and central nervous system signs, which are caused as a result of the inhibition of carboxylic esterase. Another form of neurotoxicity seen is delayed neurotoxicity, a predominant motor neuropathy, which is seen two to three weeks after exposure and is seen as a result of inhibition of a separate esterase, which is known as the neuropathy target esterase [5]. The intermediate syndrome, which manifests two to five days after an acute crisis and is marked by acute respiratory distress and skeletal muscle weakness, is the third and last kind of neurotoxicity to be observed. The persistent suppression of cholinesterase, which is unresponsive to oximes and atropine therapy, can be linked to this occurrence.

In individuals who have consumed organophosphorus pesticides, the intermediate syndrome occurs in 20% of cases and has no relationship to the type of organophosphorus pesticide. It often occurs two to four days after the initial presentation when the symptoms and signs of the acute cholinergic syndrome, such as muscle fasciculations and muscarinic signals, disappear. The intermediate syndrome is frequently characterized by weakness of the neck, proximal limb, and respiratory muscles, particularly the diaphragm, intercostal muscles, and accessory muscles. As a result, some patients may only exhibit neck muscle weakness, whereas others may experience weakness in both the neck and proximal limb muscles. Patients who already have intermediate syndrome but later develop it could need ventilator support [6].

Sometimes organophosphorus poisoning can present as myocardial infarction as reported in some case reports [7].

Wherever intermediate syndrome causes respiratory problems, immediate treatment should be given to avert mortality. Hence, in patients with organophosphorus poisoning, careful use of atropine is a must, which can work as a double-edged sword, as a high dose of atropine sometimes leads to vocal cord palsy due to superior laryngeal nerve palsy and a low dose of atropine leads to the intermediate syndrome.

## Conclusions

The neurotoxic effects of organophosphate poisoning should be considered while providing first-line care to exposed patients. Before considering isolated bilateral or unilateral vocal cord paralysis, the intermediate syndrome should be excluded if the patient develops dysphonia and respiratory distress.
